# Supplementary material for: African ancestry-enriched variants in the GATM gene are associated with elevated serum creatinine levels
Source: medRxiv. 2025 Mar 9:2025.03.07.25323581. Preprint. [Version 1] doi: 10.1101/2025.03.07.25323581 (PMC12191081; doi:10.1101/2025.03.07.25323581)
Supplement: Supplement 1 [file media-1.pdf]

Supplementary information for:

African ancestry-enriched variants in the *GATM* gene are associated with elevated serum creatinine levels

Shivam Sharma, Courtney A. Astore, Leonardo Mariño-Ramírez, I. King Jordan

Contents

Figure S1. **Analysis flowchart.** 2

Figure S2. **Serum creatinine levels.** 3

Figure S3. **Q-Q plots.** 4

Figure S4. **Conditional GWAS.** 5

Figure S5. **Conditional admixture mapping.** 6

Supplementary Table S1. ***GATM* eQTL in the Scr credible variant set.** 7

Supplementary Table S2. **Previously discovered Scr-associated variants at the *GATM* locus.** 8

Figure S1. **Analysis flowchart.** Inclusion and exclusion criteria are shown for each step along with the number of participants retained. The final cohort of 18,979 participants was used for GWAS, local ancestry inference, admixture mapping, and ancestry-specific GWAS.

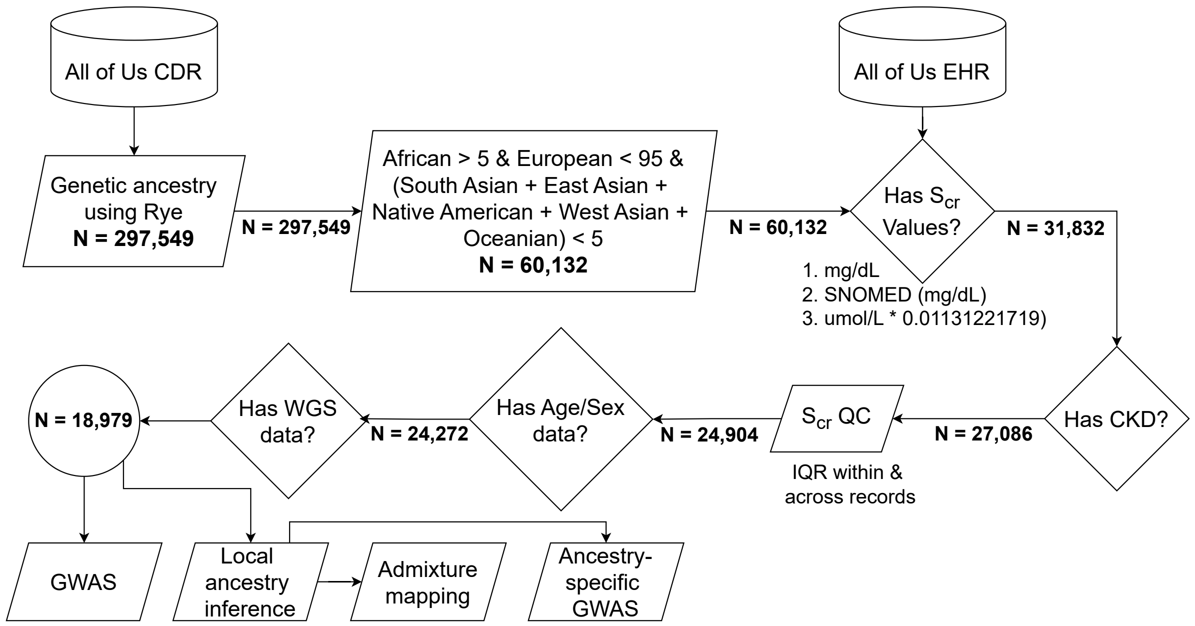

Figure S2. **Serum creatinine levels.** Distribution of serum creatinine (Scr) levels shown through histograms stratified by sex.

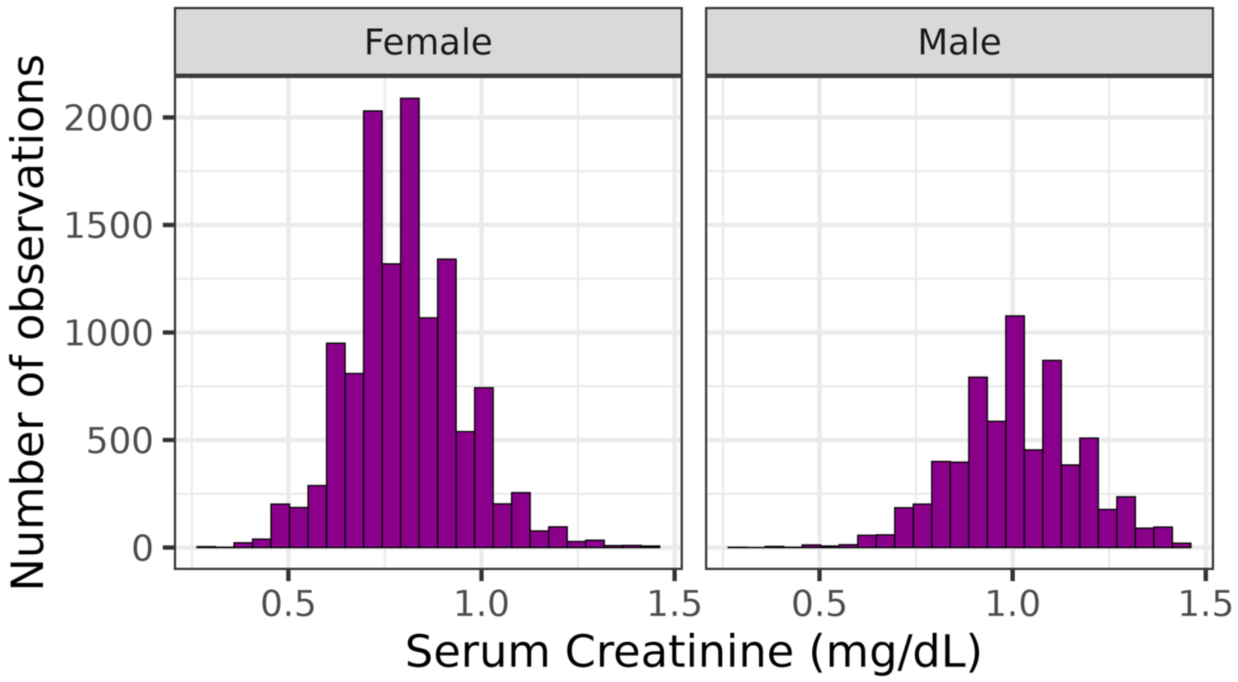

Figure S3. **Q-Q plots.** Quantile-quantile plots comparing the observed p-values vs the expected p-values for (A) standard GWAS, and (B) African ancestry-specific GWAS.

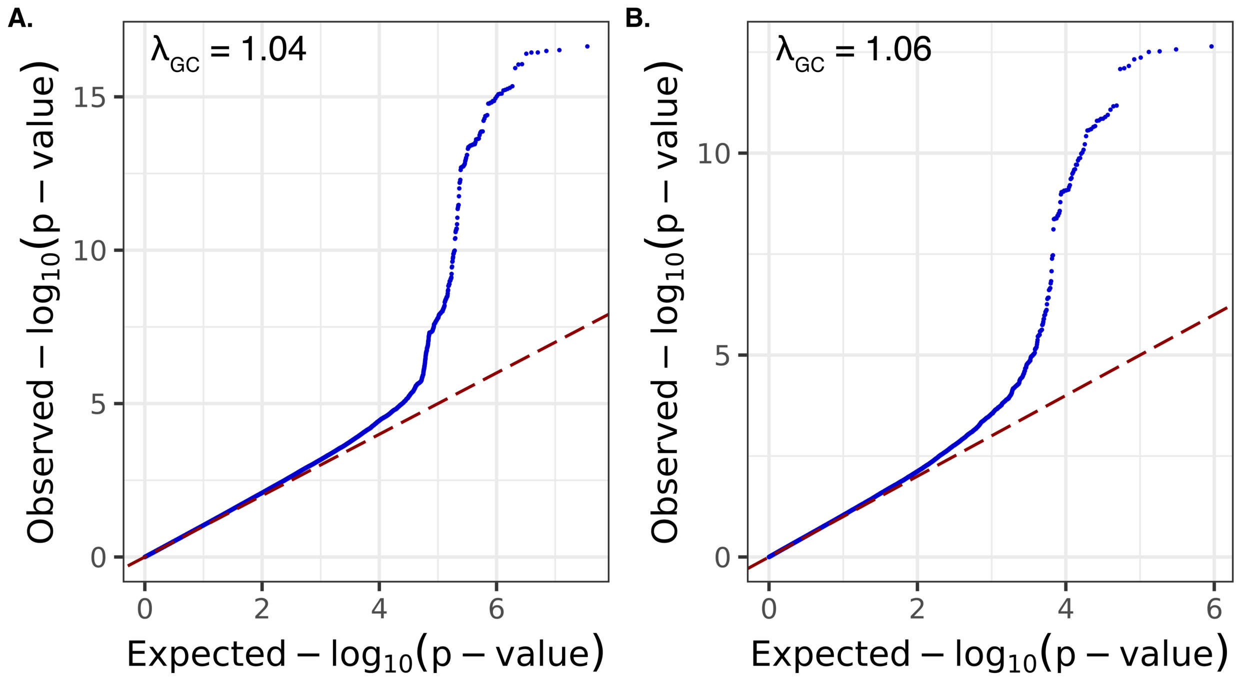

Figure S4. **Conditional GWAS.** Locus 15q23 (45.3Mb–45.5Mb), significantly associated in the standard GWAS, loses significance only when conditioned on the lead variant rs2467850, but remains significant when conditioned on the second lead variant rs55673230.

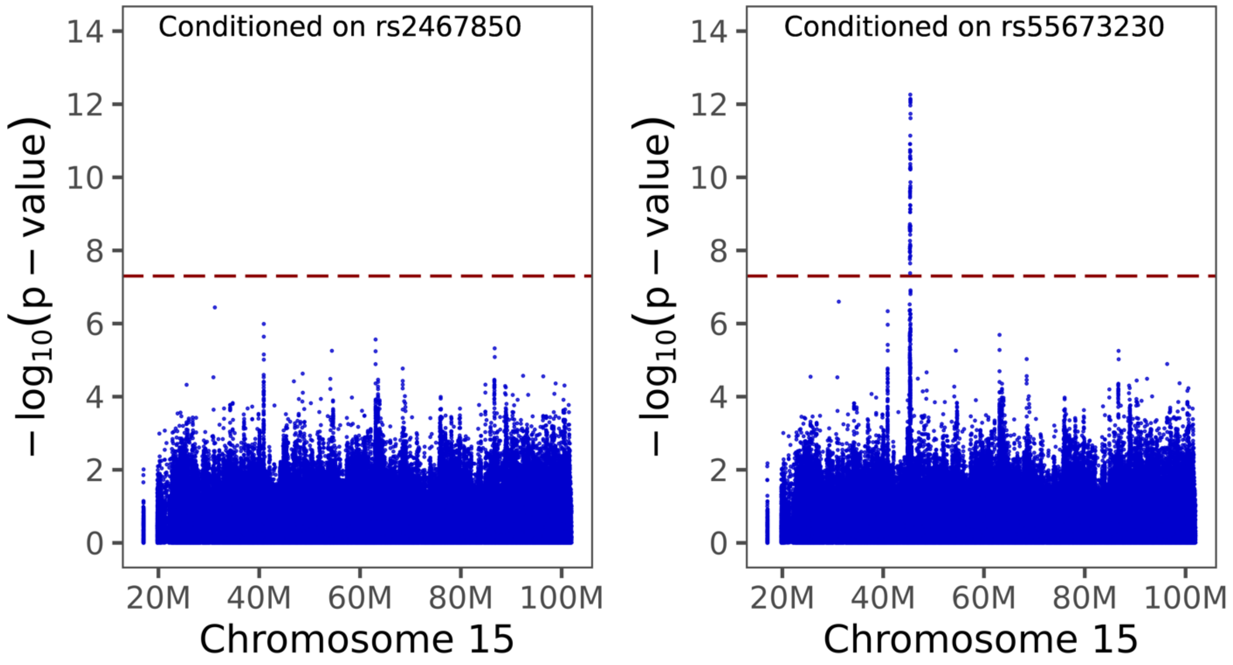

Figure S5. **Conditional admixture mapping.** Haplotypes within and around the *GATM* gene lose significance in the admixture mapping analysis when the model is conditioned on the GWAS lead variant rs2467850.

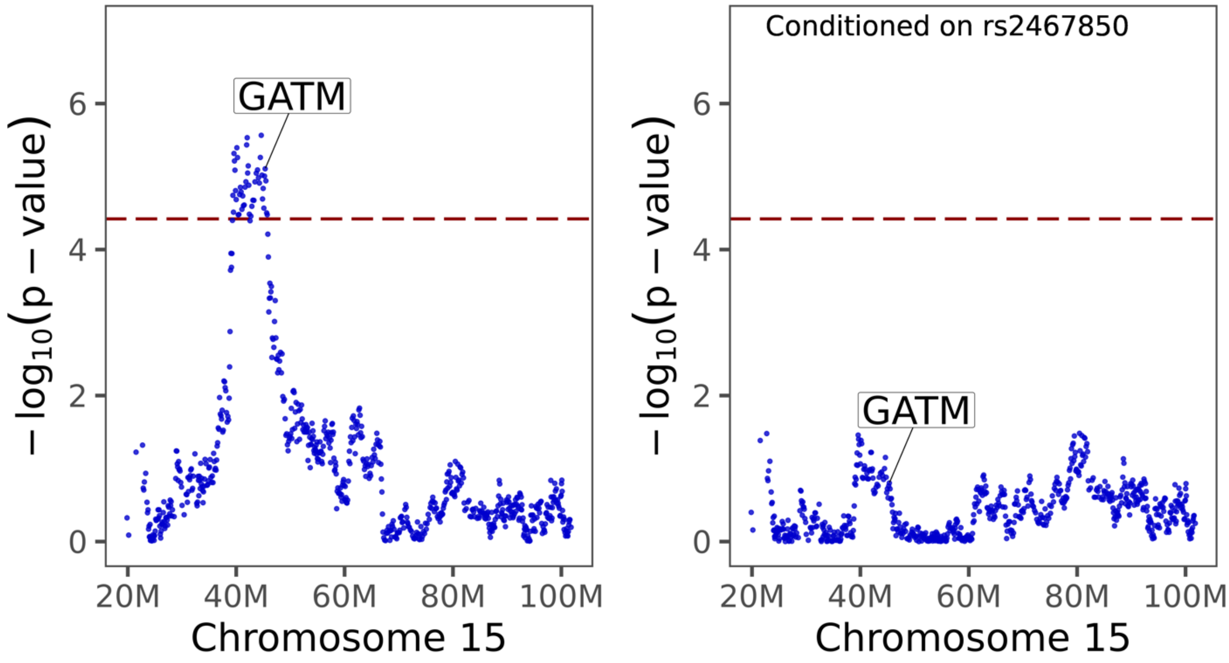

Supplementary Table S1. ***GATM* eQTL in the Scr credible variant set.** African American whole blood eQTL summary statistics for Scr credible variants. TSS dist. is the distance to the *GATM* transcription start site. AFR_{Freq} and EUR_{Freq} are the alternate allele frequencies for the Gnomad African/African American and European (non-Finnish) ancestry groups, respectively.

ID	CHR	POS	TSS dist.	REF	ALT	BETA	SE	p	AFR_{Freq}	EUR_{Freq}
rs1145096	15	45338576	39,968	A	T	0.59	0.0383	7.08×10^{-46}	0.438	0.001
rs1706802	15	45341646	36,898	G	A	0.59	0.0384	1.33×10^{-46}	0.434	0.001
rs1153856	15	45367576	10,968	T	A	0.71	0.0348	1.59×10^{-73}	0.473	0.002
rs8024550	15	45378483	61	A	C	0.7	0.0369	3.79×10^{-65}	0.424	0.001
rs2467850	15	45379909	-1,365	C	T	0.72	0.0371	1.25×10^{-67}	0.413	0.001
rs1834686	15	45402570	-24,026	G	T	0.71	0.0367	3.81×10^{-67}	0.422	0.001
rs1346265	15	45403414	-24,870	C	T	0.71	0.0351	1.06×10^{-72}	0.472	0.001
rs1153848	15	45403676	-25,132	A	G	0.71	0.0369	1.05×10^{-65}	0.414	0.001
rs2413765	15	45406220	-27,676	G	A	0.7	0.0369	9.44×10^{-66}	0.413	0.001
rs1145075	15	45407854	-29,310	A	G	0.71	0.0369	6.32×10^{-66}	0.415	0.001
rs34691692	15	45412398	-33,854	CA	C	0.72	0.0387	1.05×10^{-62}	0.434	0.002
rs2467859	15	45419421	-40,877	C	T	0.7	0.0367	1.62×10^{-65}	0.416	0.001
rs2453547	15	45425847	-47,303	C	T	0.7	0.0368	4.66×10^{-65}	0.414	0.001

Supplementary Table S2. **Previously discovered Scr-associated variants at the *GATM* locus.** Lead variants previously found to be associated with Scr or eGFR and spanning the *GATM* locus on chromosome 15 are shown along with the lead variants discovered here using standard and ancestry-specific GWAS (highlighted in yellow). The location of the *GATM* transcript start site (TSS) is shown relative to the variant positions; positive (+) distances are downstream from the TSS, and negative (-) distances are upstream from the TSS. African American whole blood eQTL summary statistics for *GATM* are shown for each variant. TSS dist. is the distance to the *GATM* transcription start site. AFR_{Freq} and EUR_{Freq} are the alternate allele frequencies for the Gnomad African/African American and European (non-Finnish) ancestry groups, respectively. Cohort ancestry abbreviations from the GWAS catalog are: AA-African American, AFR-African Ancestry, AMR-Admixed American or Hispanic, EAS-East Asian, EUR-European, NA-Native American, SAS-South Asian. PMID are the PubMed publication identifiers for the previous GWAS studies.

ID	CHR	POS	REF	ALT	AFR_{Freq}	EUR_{Freq}	TSS dist.	eQTL BETA	eQTL SE	eQTL p	COHORT ANCESTRY	PMID
rs1464559	15	45300689	A	T	0.528	0.002	+77855	0.495	0.039	5.29E-34	AA, AFR	37644460
rs2433603	15	45354028	T	C	0.495	0.002	+24516	0.618	0.037	5.51E-54	AFR	33783510
rs2461702	15	45355229	A	G	0.774	0.26	+23315	-0.184	0.052	4.38E-04	AA,AMR,EAS,EUR,NA	33418499
rs1145093	15	45357615	C	A	0.842	0.376	+20929	-0.317	0.056	1.76E-08	EUR	33414548
											EUR,EAS	31015462
rs62025168	15	45360080	G	A	0.037	0.196	+18464	0.293	0.120	1.52E-02	EUR,AFR,SAS	33462484
rs1145085	15	45365606	A	G	0.784	0.262	+12938	-0.245	0.052	2.96E-06	AA,AMR	38190104
rs1153855	15	45368560	C	G	0.843	0.376	+9984	-0.317	0.056	1.76E-08	EUR,AMR,AA	37253714
rs2486274	15	45374030	G	T	0.841	0.376	+4514	-0.314	0.055	2.15E-08	EUR	36357675
rs8024550	15	45378483	A	C	0.424	0.001	+61	0.701	0.037	3.79E-65	AA	Ancestry-specific GWAS
**GATM TSS chr15 45378544**												
rs2467850	15	45379909	C	T	0.413	0.001	-1365	0.721	0.037	1.25E-67	AA	Standard GWAS
rs2486272	15	45380055	T	C	0.789	0.272	-1511	-0.252	0.052	1.35E-06	EUR,AA	31451708
											AA,AMR,EAS,EUR	39024449
rs1145084	15	45383242	G	A	0.843	0.376	-4698	-0.321	0.056	1.13E-08	EUR	34272381
											EAS,SAS,EUR	38448586
rs1145077	15	45391597	G	T	0.843	0.375	-13053	-0.317	0.056	1.76E-08	EUR,EAS,AA,SAS,AMR	31152163
